# Mastering your fellowship: Part 3, 2025

**DOI:** 10.4102/safp.v67i1.6102

**Published:** 2025-06-03

**Authors:** Klaus B. von Pressentin, John M. Musonda, Gert Marincowitz, Selvandran Rangiah

**Affiliations:** 1 Division of Family Medicine, Department of Family, Community and Emergency Care, Faculty of Health Sciences, University of Cape Town, Cape Town, South Africa; 2 Department of Family Medicine and Primary Care, Faculty of Health Sciences, The University of the Witwatersrand, Johannesburg, South Africa; 3 Department of Family Medicine, University of Limpopo, Polokwane, South Africa; 4 Department of Family Medicine, College of Health Sciences, University of KwaZulu-Natal, Durban, South Africa

**Keywords:** family physicians, FCFP (SA) examination, family medicine registrars, postgraduate training, national exit examination, antenatal care

## Abstract

The series ‘Mastering your Fellowship’ provides examples of the question formats encountered in the written and clinical examinations, Part A of the FCFP (SA) examination. The series aims to help family medicine registrars (and supervisors) prepare for this examination. Model answers are available online.

This *South African Family Practice* journal section aims to help registrars prepare for the FCFP (SA) exit examination (Fellowship of the College of Family Physicians). It will provide examples of the question formats encountered in the written exam: Multiple Choice Question (MCQ) in the form of Single Best Answer (SBA – Type A) and Extended Matching Question (EMQ – Type R); Short Answer Question (SAQ), questions based on the Critical Reading of a Journal article (CRJ: evidence-based medicine) and an example of an Objectively Structured Clinical Examination (OSCE) question. Each of these question types is presented based on the College of Family Physicians blueprint and the key learning outcomes of the FCFP (SA) programme. The MCQs draw on the 10 clinical domains of family medicine, the SAQs align with the five national unit standards, and the critical reading section includes evidence-based medicine and primary care research methods.

This edition is based on entrustable professional activity (EPA) 2 (Managing pregnant women – antenatal care), EPA 18 (Supporting community-based health services) and EPA 22 (leading clinical governance activities). We suggest that you attempt to answer the questions (by yourself or with peers and supervisors) before finding the model answers online: http://www.safpj.co.za/.

Please visit the Colleges of Medicine website for guidelines on the Fellowship examination: https://cmsa.co.za/fellowship-of-the-college-of-family-physicians-of-south-africa-fcfpsa/.

We are keen to hear how this series assists registrars and their supervisors in preparing for the FCFP (SA) examination. Please email us (editor@safpj.co.za) with your feedback and suggestions.

## Multiple choice question (MCQ): Single best answer

A 24-year-old woman, Gravida 2 Para 1, presents to the emergency centre in the district hospital complaining of vaginal bleeding and cramping, with lower abdominal pains for 1 day. Her last normal menstrual period was 3 months ago. She attended the local antenatal clinic once, where the pregnancy was confirmed. On examination, she appears uneasy and has mild pallor. Her blood pressure is 123/72 mmHg, heart rate is 80 beats per minute, respiratory rate is 16 breaths per minute, and temperature is 37.3 °C. The cervical os is closed, there is no cervical motion tenderness or adnexal mass, the uterus feels smaller than the gestational period, and minimal blood is on the glove. The point-of-care haemoglobin is 10 g/dL, and the pregnancy test is positive. What is this patient’s most appropriate next management step?

- Assess foetal viability and advise bed rest.
- Administer misoprostol and allow for the evacuation of the uterus.
- Inform her that there is no need for further evacuation of the uterus.
- Administer misoprostol and prepare her for surgical evacuation.

Short answer: c.

### Discussion

A miscarriage is emotionally and physically distressing to the woman and her family, whether it was planned or not. Hence, health workers must approach the woman and her family respectfully and sensitively. The above-mentioned conditions are all causes of vaginal bleeding in the first trimester, which is defined as < 14 weeks gestational period. Miscarriages are common, and one in every seven pregnancies ends up with a spontaneous miscarriage. It is vital that the clinician conducts a thorough clinical assessment and excludes differential diagnoses such as an ectopic pregnancy. This assessment may be augmented by a point-of-care ultrasound if available. It is also essential to discuss a comprehensive management plan, which includes appropriate safety netting.

A *complete miscarriage* may present, as in the given scenario, with vaginal bleeding, cramping, and lower abdominal pains, with a positive pregnancy test. By the time the mother presents, the vaginal bleeding is light, the cervical os is closed, and on palpation, the uterus feels smaller than expected for the dates. The cramping and lower abdominal pain may settle soon after and no further evacuation of the uterus is needed.

However, with a *threatening miscarriage*, the woman may present with mild bleeding in early pregnancy without a dilated cervix and the uterus size corresponding to the expected dates. For diagnosis, an ultrasound scan should show a live foetus in the uterus. Treatment is conservative, and it is not necessary to admit the woman to the hospital. It is recommended that foetal viability be re-scanned and assessed if bleeding continues. Ask the woman to report back if the bleeding continues.

An *inevitable miscarriage* is the sequela of a threatening miscarriage whereby the cramping abdominal pain and vaginal bleeding worsen, much blood is lost, the cervix dilates, and intact membranes are felt. To assist in expelling the products of conception, administer 20 units of oxytocin in one litre of sodium chloride 0.9% at 125 mL/hour. Inspect the products of conception once expelled and be prepared to evacuate the uterus to remove the remaining products.

An *incomplete miscarriage* may present with cramping, lower abdominal pains, and light or heavy vaginal bleeding; the cervical os is open, with partial expulsion of the products of conception, which may be visible or felt. On palpation of the uterus, the size does not correspond with the gestation. Any visible or palpable products of conception may be removed with fingers or forceps, and the patient is observed. Should the vaginal bleeding be heavy after the removal of visible or palpable products of conception, the uterus must be evacuated. Administer oxytocin 20 units in one-litre sodium chloride 0.9% at 125 mL/hour through an intravenous line. If bleeding is mild after removal of visible or palpable products, and there are no features of unsafe miscarriage, expectant management, or treatment with 600 mcg oral or 400 mcg S/L misoprostol are options that can be offered to the patient. The bleeding should have settled entirely in 2 weeks, and the patient should return if not.

The post-miscarriage follow-up is necessary for a woman who has experienced such an event because of the distress suffered immediately, soon after and in the future. Therefore, how the woman is coping with her emotional distress is vital to manage by healthcare workers. She must be encouraged to rest, eat well, and seek help and support.

### Further reading

- National Department of Health. National integrated maternal and perinatal care guidelines for South Africa. 5th ed. [homepage on the Internet] Pretoria; 2024 [cited 2024 Dec 18], p. 50–56. Available from: https://knowledgehub.health.gov.za/elibrary/national-integrated-maternal-and-perinatal-care-guidelines-south-africa

## Short answer question: Community-orientated primary care and clinical governance in maternal health

You are the newly appointed family physician in a district hospital. The senior clinical manager requests that you work on improving the perinatal health outcomes. The stillborn rate for your sub-district is 22/1000 deliveries, and the early neonatal mortality rate is 13/100 live births. The maternal mortality rate for the subdistrict over the past year was 80/100 000 deliveries.

After a review of the indicators, you found that the antenatal booking before 20 weeks is only 45%. On reviewing the perinatal audits, you discovered that most of the stillbirths are macerated, with the most common cause being related to hypertension in pregnancy or unexplained, and most of the early neonatal deaths are because of complications of prematurity.

1. Considering the scenario, list the categories of people you want to have as part of your team. (4)
2. List four groups you would like to be part of the community health forum when addressing the problem. (4)
3. Your team is very concerned about the low antenatal booking before 20 weeks of gestation. Use the fishbone root cause analysis to identify possible causes for the low rate of early antenatal booking as part of basic antenatal care (BANC) in your sub-district and give practical examples of each. (4)
4. Based on the fishbone analysis, suggest four interventions you can implement to improve early antenatal booking. (4)
5. Mention two clinical interventions you can promote to prevent pregnancy-induced hypertension (HT) and its complications. (2)
6. Explain how you will use maternal health indicators such as BANC booking before 20 weeks, stillbirth rate (SBR), and early neonatal mortality rate (ENNMR) in your tasks given by the clinical manager. (2)
7. Hypertensive disease in pregnancy is one of your major concerns regarding the high stillbirth rate. You notice very few high-risk women were started on low-dose aspirin. What are the indications for low-dose aspirin? (3)
8. How will you ensure that the PHC system is strengthened so that more women are started on low-dose aspirin? (2)

**Total: 25 marks**

*Suggested answers (the answers should show some application to the scenario)*

1. **Considering the aforementioned, list the categories of people you want to have as part of your team. (4)**
  - Midwives
  - Doctors (including family physician)
  - Representative from the regional hospital’s obstetrics and gynaecology unit, including specialist
  - PHC nurses
  - Community health workers
  - Clinic committee members
  - Patient representatives
2. **List four groups you would like to be part of the community health forum when addressing the problem. (4)**
  - NGOs
  - Community organisations
  - Churches
  - Traditional leaders
  - Traditional healers
  - Government departments, for example social development, basic education
3. **Your team is very concerned about the low antenatal booking before 20 weeks of gestation. Use the fishbone root cause analysis to identify possible causes for the low rate of early antenatal booking as part of basic antenatal care (BANC) in your sub-district and give practical examples of each. (4)**
  - Patient-related factors, for example, beliefs and knowledge, for example, that others should not know you are pregnant as you might be ‘bewitched’ or a lack of information.
  - Social factors include poverty (costs implied by antenatal visits) and other responsibilities, such as employment, getting off from work, and looking after other children.
  - Health worker-related problems, such as attitudes and inadequate clinical expertise.
  - Health system factors include inconvenient clinic times, long queues, waiting time, and distance to the clinic.
4. **Based on the fishbone analysis, suggest four interventions you can implement to improve early antenatal booking. (4)**
  - Community health worker (CHW) door-to-door campaigns to link pregnant women with antenatal care and follow-up to ensure they are linked to care.
  - Community awareness campaigns at public meetings (community gatherings, churches, schools).
  - Encourage fathers to participate and be present in antenatal care.
  - Patient education during clinic visits.
  - Motivate clinic midwives to fast-track ANC bookings.
  - Train health workers (private and public) in current treatment guidelines, including referral for antenatal care as soon as pregnancy is diagnosed and regular screening.
  - Encourage pregnant women to register with Mom-connect.
5. **Mention two clinical interventions you can promote to prevent pregnancy-induced HT and its complications. (2)**
  - Calcium supplementation (500 mg elemental calcium daily) for all pregnant women.
  - Aspirin for high-risk pregnancies.
  - Regular BP screening during BANC plus.
  - Early treatment of hypertensive disease in pregnancy to prevent complications.
6. **Explain how you will use maternal health indicators such as BANC booking before 20 weeks, stillbirth rate (SBR), and early neonatal mortality rate (ENNMR) in your tasks given by the clinical manager. (2)**
  - Data-informed feedback and intervention planning is part of the quality improvement process.
  - It is even better if each clinic gets individual feedback on how they perform concerning these indicators to enhance further improvement planning.
7. **Hypertensive disease in pregnancy is one of your major concerns regarding the high stillbirth rate. You notice very few high-risk women were started on low-dose aspirin. What are the indications for low-dose aspirin? Mention any three. (3)**
  - Prior pre-eclampsia
  - Chronic hypertension
  - Multiple gestation
  - Pre-gestational diabetes
  - Maternal body mass index (BMI) > 35 kg/m^2^
  - Anti-phospholipid syndrome
  - Assisted reproduction therapies
8. **How will you ensure that the PHC system is strengthened so that more women are started on low-dose aspirin? (2)**
  - Develop a treatment guideline or care pathway.
  - Do in-service training and capacity building of midwives regarding these guidelines.
  - Ensure that clinical audits of maternity records are done to evaluate adherence to the guidelines.

### Further reading

- National Department of Health. National integrated maternal and perinatal care guidelines for South Africa. 5th ed. 2024 [cited 2024 Dec 18]. Available from: https://knowledgehub.health.gov.za/elibrary/national-integrated-maternal-and-perinatal-care-guidelines-south-africa
- Moodley J, Soma-Pillay P, Buchmann E, Pattinson RC. Hypertensive disorders in pregnancy: 2019 National guideline. S Afr Med J. 2019;109(9):12723.
- Marcus T, Hugo J. Chapter 7: Community-orientated primary care: Where there is a doctor. In Mash B, editor. Handbook of family medicine. 4th ed. Cape Town: Oxford University Press; 2017, p. 334–359.
- Mazaza S, Gunst C. Chapter 8: Leadership and clinical governance. In Mash B, editor. Handbook of family medicine. 4th ed. Cape Town: Oxford University Press; 2017.

## Critical appraisal of quantitative research

Read the accompanying article carefully and answer the following questions. As far as possible, use your own words. Do not copy out chunks from the article. Be guided by the allocation of marks concerning the length of your responses.

- Hlongwane TM, Botha T, Nkosi BS, Pattinson RC. Preventing antenatal stillbirths: An innovative approach for primary health care. S Afr Fam Pract. 2022;64(3):a5487. https://doi.org/10.4102/safp.v64i1.5487

**Total: 30 marks**

1. Identify three distinct arguments made by the authors to provide a rationale for the study. (3 marks)
2. Critically appraise the authors’ choice of study design to answer the research question. (5 marks)
3. Critically appraise how the authors went about with recruitment to the different cohorts. (7 marks)
4. Was the exposure measured in a valid and reliable way? (4 marks)
5. Critically appraise the strengths and weaknesses of measuring the outcomes of interest. (4 marks)
6. Comment on whether the study period selection may have introduced potential confounding factors. (3 marks)
7. Critically appraise the results concerning the study objectives, including whether the results will help locally. (4 marks)

*Suggested answers*

1. **Identify three distinct arguments made by the authors to provide a rationale for the study. (3 marks)**
  - High stillbirth rates in South Africa: Approximately 16 000 stillbirths occur annually, with 43% happening in district hospitals and community health centres (CHCs), and a significant proportion being unexplained (30% in district hospitals and CHCs).
  - Limited effectiveness of current screening methods: Existing tools such as palpation, symphysis-fundal height measurement, and routine ultrasound perform poorly in detecting foetal growth restriction (FGR), with detection rates as low as 15% – 20% in low-risk pregnancies.
  - Potential for early intervention: The study hypothesised that continuous-wave Doppler ultrasound of the umbilical artery (CWDU-UmA) could identify high-risk foetuses in low-risk pregnancies, allowing for appropriate management and potentially reducing stillbirth rates.
2. **Critically appraise the authors’ choice of study design to answer the research question. (5 marks)**
  - The researchers did not justify their choice of study design but stated that they conducted a cohort study. Their research question was to investigate whether screening a low-risk pregnant population using CWDU-UmA in primary healthcare clinics together with a standard referral protocol would result in a reduction in the stillbirth rate.
  - A cohort study design helps understand a condition’s outcome or natural history in an identified study population. As participants do not have the outcome or condition at study enrolment, this design allows for the temporal causality between exposure and outcome to be assessed.
  - This cohort study was prospective as the included participants were all recruited at the time of presentation to the non-specialist primary antenatal care clinics, and outcome data were obtained from the various delivery sites.
  - The exposure was the CWDU-UmA screening intervention, and the outcome was a reduction in the stillbirth rate.
  - Therefore, the strength of this study was that a prospective cohort study design was appropriate for evaluating screening intervention effectiveness.
3. **Critically appraise how the authors went about with recruitment to the different cohorts. [7 marks]**
  - In a cohort study, participants must be selected using mutual characteristics and grouped into cohorts based on exposure and non-exposure status.
  - In this study, the screening group (exposed to the intervention) and two control groups were recruited from the same population and recruited at primary healthcare clinics across nine sites.
  - The same inclusion criteria were used to recruit the cohorts at baseline: all candidates must be women with a singleton pregnancy, aged 18 years or more, and classified as low risk between 28 and 34 weeks’ gestation (if the gestational age was unknown, a symphysis-fundal height of 26 cm or above was used); all candidates were also required to provide written consent.
  - There were specific screening days for CWDU-UmA in the intervention group, and those not attending the clinics on those days (and therefore, not screened using CWDU-UmA) served as the control group.
  - The authors divided these controls into two groups, namely control group 1, which did not undergo the screening intervention and control group 2, which consisted of a subset of control group 1 but excluded those who subsequently developed antenatal complications.
  - The rationale for these two control groups was that the authors wished to ensure that the screened population was ‘as low-risk a population as possible’. The authors aimed to measure the effectiveness of the screening intervention in reducing the stillbirth rate in women classified as having low-risk pregnancies.
  - Limitations include the non-randomised recruitment, which was used for budgetary reasons. Screening was performed only on specific days, potentially introducing selection bias. These limitations may explain potential confounding because of differences in group characteristics (e.g. more nulliparous women in the study group), as described in the results section.
4. **Was the exposure measured in a valid and reliable way? (4 marks)**
  - Yes, the screening intervention exposure was measured validly. Participants in the intervention group were screened using an Umbiflow^TM^ device linked to a flow diagram as informed by previous studies by the same authors.
  - Resistance index (RI) results were classified as normal or abnormal based on 75th centile cut-off.
  - Women with abnormal RI were referred to high-risk clinics as per protocol and followed up weekly or fortnightly with Doppler ultrasound and growth scans.
  - One could argue that the article did not provide the detail on ensuring consistent and reliable measurement using the device. The authors did state that ‘the different sites started at different times to allow for adequate training and quality control at all of the nine sites’. The authors cited a separate article in which they presumably described the training and quality control details.
5. **Critically appraise the strengths and weaknesses of measuring the outcomes of interest. (4 marks – 2 strengths and 2 weaknesses)**
  - It is important to assess the objectivity of the measurements and whether the measurement methods were applied similar to the different cohorts to look for measurement or classification bias. One should also consider whether the outcomes were measured validly and reliably and whether the follow-up time reported is sufficient to allow outcomes to occur.
  - Strengths:
    ■ Electronic birth registers were used to collect outcome data.
    ■ Defined clear outcome measures (stillbirth rate, small for gestational age).
    ■ World Health Organization (WHO) foetal growth charts were used to categorise birth weight.
    ■ Collected comprehensive clinical information at enrolment.
  - Limitations:
    ■ Single screening point (28–34 weeks gestation) – it could be possible that the RI value may have been different if screened at a different date.
    ■ Incomplete tracing of neonatal outcomes, as this information was not captured in the electronic birth registers and the Perinatal Problem Identification Programme dataset did not contain the granular level detail of clinic attended for antenatal care.
    ■ Reliance on the existing electronic birth register at the various delivery sites (potential for inconsistent data capturing by clinical staff).
6. **Comment on whether the study period selection may have introduced potential confounding factors. (3 marks)**
  - Data collection of the screened women and delivery outcomes started in September 2017 and stopped in February 2020, allowing time for the pregnant women to deliver by the end of February 2020.
  - Potential confounding factors include (name any 3):
    ■ Screening only on specific days might introduce selection bias
    ■ Variability in healthcare provider skills and device usage
    ■ Differences in referral and management protocols across sites
    ■ Seasonal variations in healthcare access
    ■ Potential socioeconomic differences not fully accounted for
7. **Critically appraise the results concerning the study objectives, including whether the results will help locally. (4 marks)**

*General guidance (not part of the model answer):*

- *One needs to consider the study’s results and how these have been reported, including the strengths of the association between exposure and outcome, such as risk reduction.*
- *It is important to assess the results in relation to the study design and methods:*
  ■ *One observational study rarely provides sufficiently robust evidence to recommend changes to clinical practice or within health policy decision-making.*
  ■ *For certain questions, observational studies provide the only evidence.*
  ■ *Recommendations from observational studies are always stronger when supported by other evidence.*

*Suggested answer:*

- This study built on a previous study in Mamelodi township in which the same screening intervention resulted in reducing the stillbirth rate in women classified as having low-risk pregnancies by 43%. The authors now intended to investigate whether screening a low-risk pregnant population using Umbiflow^TM^ in primary healthcare clinics throughout South Africa, together with a standard referral protocol for foetuses with abnormal RI, would result in a reduction in the stillbirth rate.
- This study was conducted in a national frame and included nine study sites across eight provinces in South Africa. The dataset included 17 368 pregnancies. The study successfully demonstrated a significant reduction in stillbirth rates (35% – 43%) through CWDU-UmA screening, supporting the primary objective of identifying high-risk foetuses in low-risk populations and implementing targeted management.
- The authors described some study limitations, including their decision not to randomise women so that a larger sample of women could be recruited in a shorter period for budgetary reasons. There was also some degree of loss to follow-up, but the authors argued that the unknown outcomes of only 7.8% are particularly good for low-income and middle-income settings where women are considerably more mobile and relocate frequently.
- In summary, the results will help locally, provided that more research is performed to understand better the timing of the screening intervention during the antenatal care period and how best to scale up the training and access to the screening equipment. The implications on specialised care services planning should also be considered, especially as increased screening could result in increased referrals and more pressure on existing maternity and neonatal high care and intensive care resources.

### Further reading

- Pather M. Evidence-based family medicine. In Mash B, editor. Handbook of family medicine. 4th ed. Cape Town: Oxford University Press, 2017, p. 430–453.
- The Critical Appraisals Skills Programme (CASP). CASP cohort study checklist [homepage on the Internet]. 2024 [cited 2024 Dec 06]. Available from: https://casp-uk.net/casp-tools-checklists/
- JBI. Critical appraisal tools – Checklist for cohort studies [homepage on the Internet]. 2024 [cited 2024 Dec 06]. Available from: https://jbi.global/critical-appraisal-tools
- Capili B, Anastasi JK. Overview: Cohort study designs. Am J Nurs. 2021;121(12):45. https://doi.org/10.1097/01.NAJ.0000803196.49507.08

## Objectively Structured Clinical Examination (OSCE) station scenario

*Objective:* This station tests the candidate’s ability to facilitate informed consent for a surgical procedure (elective caesarean section and tubal ligation).

*Type of station:* Integrated consultation.

*Role players:* Female patient.

### Instructions for candidate

- You are allocated to the obstetrics department of the local district hospital.
- Mrs TB, a 36-year-old Gravida 5, Para 4 (four living children), presents for admission for an elective caesarean section and tubal ligation procedure. It will be performed under spinal anaesthesia.
- Your task: Conduct a focused consultation with this patient to obtain informed consent for the caesarean section and tubal ligation procedure.

### Instructions for the examiner

- This is an integrated consultation station in which the candidate has 20 min.
- Familiarise yourself with the assessor guidelines, which detail the expected responses from the candidate.
- No marks are allocated. In the mark sheet (Table 1), tick off one of the three responses for each competency listed. Ensure you are clear on the criteria for judging a candidate’s competence in each area.

**TABLE 1:** Marking sheet for consultation station.

Competencies	Candidate’s rating		
	Not competent	Competent	Good
Establishes and maintains a good doctor–patient relationship	-	-	-
Gathering information	-	-	-
Clinical reasoning	-	-	-
Explanation and Planning	-	-	-
Creates a structure and flow to the consultation	-	-	-

### Guidance for examiners

A working definition of competent performance is when the candidate effectively completes the task within the allotted time in a manner that maintains patient safety, even though the execution may not be efficient and well structured.

- *Not competent:* patient safety is compromised (including ethically and legally) or the task is not completed.
- *Competent*: the task is completed safely and effectively
- *Good*: In addition to displaying competence, the task is completed efficiently, empathically, and patient-centred (acknowledges patient ideas, beliefs, expectations, concerns, or fears).

1. Establishes and maintains a good doctor–patient relationship:

**Competent candidate:** Establishes rapport within an ethical framework, especially regarding respecting the patient’s autonomy. Introduces self and explains the purpose of the consultation. Confirms patient’s identity; verifies the operation to be done; and explains his or her role and purpose of the encounter.

**Good candidate:** In addition, do this with confidence so that the patient is seen to respond well to the candidate’s efforts. Recognises that obtaining informed consent is an interactive process, not simply receiving a signature on a form.

2. Gathering information:

**Competent candidate:** Identifies current medical issues and any bio-psychosocial risks related to the procedure. Elicits the patient’s baseline understanding of the reasons for the surgery and procedure and obtains the patient’s familiarity and experience with general and spinal anaesthesia. The understanding must include the surgery and procedure, why they are being performed, alternatives or no alternatives, principal benefits and risks, and consequences.

**Good candidate:** Explores the patient’s illness experience, fears and expectations, help-seeking behaviour, and context in terms of arriving at this point of requiring surgical intervention. The candidate actively seeks to understand the values and belief systems specific to this patient. The candidate supports the patient’s decision, even if it goes against the candidate’s beliefs. The candidate addresses disagreements with respect and empathy.

3. Clinical reasoning:

**Competent candidate:** Determines the patient’s capacity to understand and deal with the information provided to make a decision. This is a requirement to assess that the patient is legally competent, that no contraindications exist and that he or she is aware of the risks attached to the procedure. Also, considers possible alternatives to the procedure and anaesthetic.

**Good candidate:** Makes a comprehensive assessment, which is discussed and reviewed with the patient.

4. Explaining and planning:

**Competent candidate:** Clearly explains the procedure and all potential risks and benefits. Avoids medical jargon and considers the patient’s level of literacy. Discusses the team members involved and mentions the possibility of emergency interventions if things go wrong (e.g. blood transfusion). Checks back about the patient’s understanding of the operation to be performed and responds to any additional questions. Facilitates an uncoerced decision by the patient as to whether he or she agrees with the procedure after consideration of all the information that the candidate provides. Completes their sections of the legally valid consent form and assists the patient in completing their parts of the form correctly. Should the patient not be deemed legally competent, makes a secondary plan to obtain consent from the appropriate person or entity.

**Good candidate:** The candidate checks what the patient already knows and adds to the patient’s knowledge. The candidate may also use visual aids to explain the procedure and complications to the patient. The candidate must inform the patient that she does not need to make an immediate decision and can change her mind later. The patient must retain the information for a reasonable period to weigh the balance and make a reasoned decision. The candidate asks the patient to repeat in their own words what they understand by the consent, the anaesthetics, and the surgery.

5. Creates a structure and flow to the consultation:

**Competent candidate:** Ensures a structure and rational flow to the consultation to the point of obtaining (or not) the patient’s signature on the form. The form is made visible in the recording and is complete.

**Good candidate:** The candidate also appears comfortable with a non-linear flow to the consultation, integrating and prioritising the sequence to make the consultation easy, yet comprehensive. The candidate does not rattle through a recipe of ‘must-dos’ but demonstrates an easy conversation in which an expert will recognise the structure and reasoning.

### Role play – Instructions for the female patient

You are a 36-year-old woman.

**Appearance and behaviour:** Generally well, but worried about having the procedure – but determined to go through with it – and will consent once your questions have been answered.

**Opening statement:** Hi Doctor, I’m here for my C-section and sterilisation operation later today.

**Open responses: Freely tell the doctor:**

**You have four children – youngest is 2 years old – others are 10, 7, 4 years – your family is complete**

A nursing sister at the family planning clinic suggested you do sterilisation when having the C-section

**Closed responses: Only tell the doctor if asked …**

**Ideas –** you know that this operation is 100% effective.

Your husband does not want to use condoms and neither of you wants another child. He is refusing vasectomy.

You’ve explored oral and injectable contraception, but they gave you side effects: headaches and weight gain.

**Concerns –** very anxious. Your last experience of caesarean section (last baby – labour was too slow) was traumatic due to wound infection and the emergency situation. You had spinal anaesthesia. You have never had general anaesthesia.

**Expectations –** good care after the operation to prevent wound infection. You want to be able to take care of the new baby.

**Medical history:** Fit and healthy and no chronic medication. Taking pregnancy supplements (2 different tablets)

**Gynaecological history:** The 10, 7, and 4-year-old children delivered normally; the youngest, 2 years old, by caesarean section. The patient has a normal menstrual cycle. Last Normal Menstrual Period (LNMP): Can’t remember … The doctor says you are 39 weeks.

**Family and social history:**

You drink occasionally – at family functions only – 3–4 times/year (not while pregnant); no smoking.

The family history is negative for any other conditions.

Your husband is fearful for you because of your last surgical experience, but you are determined not to have another baby after this one. He supports your decision.

You are a teacher – employed at a local primary school – maternity leave

The husband will be the main caregiver to the children while you are in the hospital – assisted by his sister. They will both stay at home until you are fully recovered. Will 1 week be enough?

Support structures: Your parents have died; you were an only child; however, you have one good friend, a teacher-colleague from school, who is on standby in case she is needed.

### Patient’s notes

Gestational age: 39 + 3 weeks by early ultrasound

Booking blood test results: rapid plasma regain (RPR) negative, Rhesus (Rh) positive, HIV non-reactive, Hb:13 g/dL

Blood pressure: 118/75 mmHg

Heart rate: 90 beats per minute

Temperature: 36.4 degrees Celsius

Urine dipsticks: no abnormalities detected

Clinically stable and foetal movements are present

### Further reading

- Medical Protection Society. Consent – The basics. Factsheets. Medical Protection Society homepage [homepage on the Internet]. 2021 [cited 2024 Dec 18]. Available from: https://www.medicalprotection.org/southafrica/casebook-and-resources/factsheets/factsheets/sa-consent-the-basics
- HPCSA. Booklet 4: Seeking patients’ informed consent: the ethical considerations. Guidelines for good practice in the healthcare professions. HPCSA homepage [homepage on the Internet]. 2021 [cited 2024 Dec 18]. Available from: https://www.hpcsa.co.za/Uploads/professional_practice/ethics/Booklet_4_Informed_Consent_vDec_2021.pdf

